# Environmental Restrictors to Occupational Participation in Old Age: Exploring Differences across Gender in Puerto Rico

**DOI:** 10.3390/ijerph120911288

**Published:** 2015-09-10

**Authors:** Elsa M. Orellano-Colón, Gail A. Mountain, Marlene Rosario, Zahira M. Colón, Sujeil Acevedo, Janiliz Tirado

**Affiliations:** 1 School of Health Professions, Occupational Therapy Program, Medical Sciences Campus, University of Puerto Rico, P.O. Box 365067, San Juan 00936-5067, Puerto Rico, USA; E-Mails: marlene.rosarioabreu@upr.edu (M.R.); zahira.colon2@upr.edu (Z.M.C.); sujeil.acevedo@upr.edu (S.A.); janiliz.tirado@upr.edu (J.T.); 2 Health Services Research, University of Sheffield, Sheffield S10 2TN, UK; E-Mail: g.a.mountain@sheffield.ac.uk

**Keywords:** engagement in occupation, participation, environment, older adults, gender differences

## Abstract

Many older adults face challenges that prevent them from accomplishing common daily activities such as moving around, home maintenance, and leisure activities. There is still a need to examine and understand how environmental factors impact daily participation across gender. This study sought to make a qualitative comparison of gender differences regarding environmental barriers to participation in daily occupations from the perspectives of older adults who live alone in Puerto Rico. Twenty-six Hispanic older adults, 70 years or older participated in this study. We used a descriptive qualitative research design in which researchers administered an in-depth interview to each participant. The results elucidated that women were more likely than men to experience restricted participation due to lack of accessibility of the built environment and transportation systems. The findings could help with the development of tailored, occupation-based, preventive interventions that address gender specific environmental barriers and promote greater participation among both women and men. Further research is required to explore whether these environmental barriers to occupational participation remain consistent across living situations, socioeconomic status and ethnicity.

## 1. Introduction

Considerable theoretical evidence exists to support the interconnectedness between participation in meaningful occupation and health [1,2,3]. Engagement in meaningful occupation not only meets biological needs but also is essential for healthy adaptation, meeting intrinsic needs and interests, and for promoting wellbeing [2,4]. Moreover, the Person Environment Occupational Performance (PEOP) Model, proposes that participation in meaningful occupations, defined as goal directed activities of daily life that are specific to the individual, is essential to maintaining of health, wellbeing, and quality of life [1]. The ability to participate in occupations requires a complex interaction between the individuals’ characteristics (including psychological/emotional factors, cognition, neurobehavioral, physiological and spiritual factors) and the environmental factors (including social support, societal systems, policies and attitudes, natural and built environments, and cultural norms and values). Thus, to achieve a desired level of participation, people and groups require the support of personal as well as environmental enablers and must overcome barriers that limit their participation in activities, tasks, and roles that are important and meaningful to them. Since participation is always influenced by the characteristics of the environment in which it occurs, optimal levels of participation requires attention beyond the typical focus on the personal level, to address the broader community environmental restrictors and enablers. 

Research has shown that a supportive environment can influence older people wellbeing. For example, built environmental factors, such as high walkability and good access to parks have been associated with better overall mental health compared to less positive environmental attributes [5,6]. Therefore, the accessibility provided by the physical environment is important in supporting individual’s performance of occupations, thus resulting in a positive health impact. The natural environment, which includes geographical features such as terrain, hours of sunlight, climate, and air quality, can also influence a person’s occupational performance and health [1]. For example, climate factors can create occupational requirements (driving on a rainy day) that influence necessary tasks, required capabilities, and comfort. Social support and participation in social networks have been found to support wellbeing and active ageing, as well as limit cognitive and physical decline in of older people [7,8,9,10]. 

Moreover, social and economic systems including economic conditions and availability of resources determines whether or not an individual can participate in necessary or meaningful activities [1]. Previous studies have reported that satisfaction with community services and good quality of neighborhood facilities enabling successful occupational engagement are associated with better health [11,12]. Similarly, higher levels of education and good economic conditions of older people have been found to be protective factors against depression and physical function limitations [13].

On the other hand, environmental factors may also play a role in restricting occupational participation, and thus, the health of vulnerable groups of older people [1]. This article discusses environmental factors that result in occupational challenges related to gender. For the purpose of this study, we used the domain of the environment as defined by the PEOP model. Environmental factors refers to the external characteristics of the person that influence participation in daily occupations, including social support, social and economic systems, culture, the built environment, and the natural environment [1]. Occupational challenge is defined as any restriction to achieving a desired level of participation in meaningful occupations. The experience of challenges to participate in daily occupations is critical, as occupational participation is considered important for healthy aging [14]. Recognizing diversity in the experience of challenges to occupational participation, including gender differences, is vital in ensuring the health of both men and women as occupational beings. Whilst some progress has been made in this area, previous research studies have failed to understand gender differences in the experience of occupational challenges from an occupational perspective. One way to focus on how engagement in occupations is understood is by studying the environmental factors that results in challenges for engaging in occupations from the perspective of the participants. Therefore, the purpose of this study was to make a qualitative comparison of gender differences regarding environment related factors that results in challenges to participation in daily occupations from the perspectives of older adults who live alone in Puerto Rico. Knowledge of the occupational participation challenges facing Puerto Rican older adults, related to gender, would then advance understanding of cultural and environmental contexts that result in barriers for engaging in occupations. This new knowledge will help occupational therapy practitioners with specific cultural knowledge, design interventions that overcome these barriers and support healthy ageing at both the individual and societal levels. What follows is a description of environmental factors restricting participation of older adults who live alone, gender related factors restricting occupational participation of older people, and environmental constraints faced by older Puerto Ricans. 

### 1.1. Living Alone, Environmental Restrictors, and Occupational Challenges 

Older adults with reduced social network, such as those who live alone, as compared to those who live with others, may experience increased barriers to access the environmental support and resources required to participate in occupations that are necessary and meaningful to them. For example, a secondary data analysis was conducted from a randomized controlled trial that surveyed 2641 community-dwelling non-disabled people aged 65 years and over in the UK [15]. The findings from this study revealed that those who live alone reported higher risk of social isolation as compared to those who live with others. Similarly, another study using population-based data from the National Social Life, Health and Aging Project found that social disconnectedness and perceived isolation experienced by older people who live alone was also a barrier to engagement in daily occupations [16]. 

Those who live alone may also experience additional environmental restrictors to participate in daily life occupations. For example, a study conducted with well older people who live alone in California, revealed that limited access to safe transportation and costs associated with resources where environmental barriers to social participation [17]. Disparities in the availability of social and community resources deny older adults who live alone the opportunities to engage in meaningful occupations in a wide variety of ways including ability, motivation, and available resources. When older adults experience diminished opportunities and resources that enable them to participate in the desired range of meaningful occupations, they experience occupational injustice [18]. Since occupational injustice occurs when people are restricted in their participation in occupations to meet their basic needs and experience wellbeing [19], older adults living alone may be a population vulnerable to occupational injustice as a result of environmental constraints.

### 1.2. Gender, Environmental Restrictors and Occupational Participation 

Knowledge of gender differences in environmental factors that restrict engagement in health promoting occupations is scarce. Data from 1607 older adults in Germany who took part in a seven year follow-up telephone interview revealed that lack of societal resources for participation in sports or leisure activities and lack of transportation were barriers to participation in physical activity occupations [20]. Women reported these barriers more frequently than men, but this study failed to explore the reasons for these differences. In a cross-sectional study conducted with 127 older adults from the U.S., women reported poorer health and greater structural barriers to involvement in community-based senior activities as compared to men [21]. Moreover, higher risk of social isolation for women living alone as compared to their men counterpart has also being reported in a previous study [15]. These studies reveal some differences in environmental and societal factors that restrict participation in health promoting occupations of daily living. Still, none of these studies have examined the experiences of Hispanic older adults living alone across gender. None of these studies have systematically studied the barriers to participate in daily activities from an occupational science perspective. Therefore, a study based on the perspectives of men and women will improve the understanding of how environmental factors interact and are experienced as challenges across gender. This new knowledge will help occupational therapy practitioners to develop culturally sensitive and contextually relevant occupation-centered interventions for men as well as for women. 

### 1.3. Socioeconomic Risks for Occupational Participation 

Older Puerto Ricans who live alone experience social and economic conditions that may threaten their opportunities to engage in meaningful occupations. For example, national data from a representative sample of the Puerto Rican population revealed that 49.5% of people 65 years and older have an educational level less than high school and 46.0% have an annual income less than $10,000 [22]. Although 98.0% of the sample reported having health insurance, limited access to health care facilities and low quality of health care constitute contextual barriers that may hinder opportunities to engage in health management and maintenance occupations. A national survey revealed that older Hispanics, 65 years and older living in Puerto Rico, reported a twofold higher rate of having difficulty doing errands alone such as visiting a doctor’s office or shopping (29.9%) as compared to older adults living in the United States (15.8%) [23]. Therefore, environmental factors affecting older Puerto Ricans such as low income levels, low educational attainment, and limited access to health care, may create an unsupportive environment that may hinder this population’s opportunity to participate fully in all desired, meaningful occupations. 

In summary, gender differences and environmental factors are determinants of participation in daily activities. However, knowledge related to gender differences on the experience of environmental factors resulting in occupational challenges is scarce. The exploration of gender differences in the experience of occupational challenges in understudied populations at risk for occupational restrictions is important to understanding environmental barriers to good health. Socio-economically disadvantaged Hispanic older adults living alone in Puerto Rico is one group that has received little attention. New knowledge related to gender differences can better be gained when considering environmental factors that impact participation in daily life occupations [1].

## 2. Experimental Section 

All subjects gave their informed consent for inclusion before they participated in the study. The study was conducted in accordance with the Declaration of Helsinki, and the protocol was approved in 19 January 2012 by the Institutional Review Board of the University of Puerto Rico, Medical Sciences Campus (A4120111). We used a descriptive qualitative research design guided by the environmental domain of the PEOP model [1] to gain in-depth understanding of gender differences on the experience of occupational challenges from the perspectives of participants of this study. Descriptive qualitative research was the most suitable method for this study because its goal is to provide a comprehensive understanding of specific events from the perceptions of people who experience those events [24]. We conducted individual in-depth interviews with the participants to explore gender differences on the experience of environmental factors resulting in occupational challenges as perceived by older adults who live alone in Puerto Rico. The specific event of interest to this study was the experience of environmental restrictions to participation in daily life occupations. For the purpose of this study, we conceptually defined daily life occupations as the range of activities of daily living, instrumental activities of daily living, social activities, rest, and sleep activities that were restricted by environmental factors. 

### 2.1. Recruitment Procedures

We posted flyers in locations frequently visited by older adults, such as senior centers, churches, and doctors’ offices. If interested, individuals were asked to call the Principal Investigator (PI) to determine their eligibility for the study. If deemed eligible, an appointment was then scheduled for administration of the study’s assessment tools at a location of individual’s choosing (*i.e.*, their home, the PI’s office). We recruited four participants using flyers. Through snowball sampling procedures, we recruited an additional 22 participants. In this procedure, the researchers asked previous participants who agreed to participate in the study to make an initial contact with someone they knew who might be willing to participate. If this person was interested in participating in this study, they were asked to call the researcher to learn more about the study, determine their eligibility, and set up an appointment for an interview in their location of preference. None of the recruited participants refused to participate.

### 2.2. Participants

We recruited a purposive sample of 26 Hispanic adults (14 women and 12 men) 70 years and older who lived alone in the urban metropolitan area of Puerto Rico. We defined Hispanic as Spanish speaking adults. The selection of 26 participants was determined after reaching the point in which no new or relevant information emerged with respect to the experience of occupational challenges, and were therefore saturated in each gender group. 

Inclusion criteria included: (1) Hispanic men or women age 70 years and older; (2) living alone at home in an urban community of the metropolitan area of Puerto Rico; (3) not receiving home health care services; (4) willing to participate in this interview in the participants location of preference; and (5) having preserved cognitive function evidenced by a score of 12 and above in the Caban Minimental [25]. Participants 70 years and older were included because functional limitations to participate in daily activities increases with age. Older adults not receiving home health care services were included because we wanted to recruit individuals with no significant functional limitations to focus our sample on those older people who are still independent but approaching the peak of transitioning into dependence. Non-Hispanic older adults were excluded because their occupational participation patterns are culturally different compared with Hispanic older adults. Participants with significant cognitive issues were excluded to recruit older adults able to engage in an in-depth reflection required by the interview process 

Recruited men and women participants were similar to each other in terms of age and most were living below poverty levels (see Table 1). However, women reported a higher number of health conditions and also had higher educational levels.

**Table 1:** Participants’ characteristics.

Characteristic	Men (*n* = 12)	Women (*n* = 14)
Age range (years)	70–94	70–92
Age (mean, SD)	80.15 ± 7.4	78.9 ± 5.6
Below Poverty Levels ^a^ (%, n)	92 (11)	86 (12)
Education level (%, n)		
High school or less	90 (18)	43 (6)
Some college education	2 (10)	8 (57)
Health Conditions (%, n)		
Musculoskeletal	67 (8)	17 (1)
Cardiovascular	28 (2)	29 (4)
Diabetes	33 (4)	29 (4)
Hypertension	50 (6)	50 (7)
Osteoporosis	0	29 (4)
Rheumatoid Arthritis	0	50 (7)

^a^ Based on the federal poverty guidelines of 2015 of $11,770 total yearly income for one person in household set by the United States Department of Health and Human Services [26].

### 2.3. Data Collection Procedures

The PI and three occupational therapy graduate students trained by the PI administered the study’s measures. The first step involved administration of the *Screening Questionnaire* during the first telephone contact with the participant to assess eligibility on the basis of the first four inclusion criteria. Afterwards, an individual face-to-face meeting was arranged with all who were eligible. During this meeting, interviewers provided participants with a full explanation of the study and reviewed the consent form to ensure that all questions were answered and that their participation was voluntary. The information provided included the purpose of the study, procedures, participant’s right to stop the interview or withdraw permission for tape recording at any time, and how confidentiality would be assured. Participants understanding of the study was assessed by their responses to questions such as: ‘Can you summarize what is written in the consent form?’, ‘Can you describe what the questions in the interview are about?’ and ‘Can you tell me how long do we expect you to participate?’After addressing the participant’s questions, all subjects agreed to participate in this study and signed the consent form themselves with interviewers signing as witnesses. Afterwards, the participant was given a copy of the signed consent form and the Caban Minimental examination was administered. This was the last eligibility criteria to determine cognitive ability. All screened participants obtained the cut off score of 12 or above on the Caban Minimental examination, indicating the absence of marked cognitive impairment. Participants were then asked to complete a paper-based socio-demographic questionnaire developed for the purposes of the study. Finally, the in-depth interview was conducted during the same meeting. The interviews lasted between one and two hours and were conducted in the participant’s site of preference, such as their home or local coffee shop. The interviewers digitally recorded each interview and an independent transcriber prepared verbatim electronic text transcriptions of audio-recorded interviews for subsequent analysis. Each participant was assigned a coded number to preserve participant anonymity. Transcriptions and data banks were destroyed upon completion of this study. All interviews, transcriptions and data analyses were conducted in Spanish and translated into English for publication purposes.

### 2.4. Data Collection Instruments

*In-depth interview.* The research team designed the interview guide (Supplementary File), following the PEOP model, consisting of five open-ended questions. Questions addressed participant-perceived meaningful occupations, and in particular, the difficulties, barriers or obstacles that might restrict their participation in daily life occupations. Prompts were used to assist in focused elaboration and depth in participants’ responses related to specific environmental factors resulting in the experience of occupational challenges. The guide was also structured to capture information through field notes about participants’ enthusiasm, body language, and possible themes in the responses to the key questions. 

### 2.5. Methods of Data Analysis

The PI and three occupational therapy graduate students analyzed the qualitative data from transcribed interviews using a rigorous content analysis [27]. Content analysis is a useful approach when the purpose is to classify and summarize descriptive qualitative data. We used a theory-driven approach to categorize the codes within the conceptual organization of the environment as described by the PEOP model [1]. By using this approach, the PI and each coder began by conducting their own data analysis of the field notes and the interviews with each reviewer identifying the recurrent categories that gave meaning to the data within the environment domains of *built environment barriers*, *natural environments barriers*, *social and economic systems barriers,* and *social interaction barriers* to develop the initial coding scheme of significant statements. Afterwards, the PI, as well as the three independent coders, met four times to compare their initial coding scheme, discuss discrepancies and establish inter-coder agreements, resulting in the recodification of the data into major themes and sub-themes within the environment domains of the PEOP model. Finally, the PI and the three coders developed definitions of the resulting themes and subthemes. We assessed data trustworthiness by using investigator triangulation and transferability techniques. Investigator triangulation was employed by using the PI and the three independent coders to investigate the recurrent categories that resulted in the environmental restrictors to occupational participation across gender. This method of triangulation brought different perceptions of the inquiry and helped to strengthen the integrity of the findings. The transferability technique was employed through the use of a purposeful sample and a provision of a thick description of the study methods and participants. NVivo software (Version 9) was used as a data manager and organizer.

## 3. Results 

**Figure 1:**
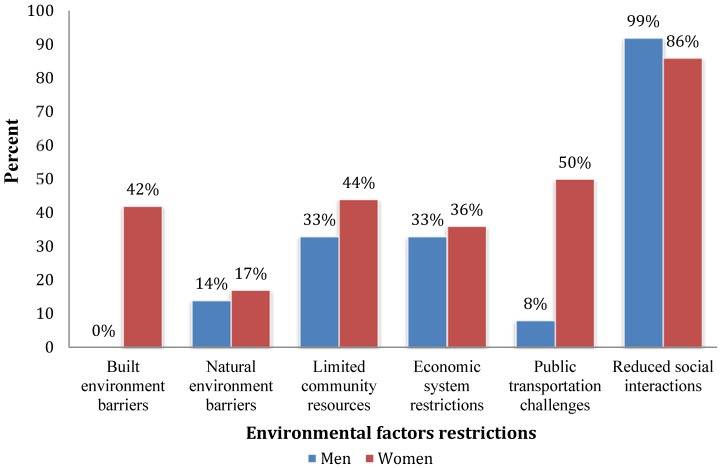
Percent of environmental factors restrictors to occupational participation of men and women.

The results were grouped according to the extrinsic factors of the environment or community conditions identified from data when participants talked about aspects of their community that negatively affect their ability to engage in meaningful occupations at home or at the community level. Themes include: *built environment barriers*; *natural environments barriers*; *social and economic systems barriers;* and *social interaction barriers.* Some of these themes where further expanded into subthemes. We compared these themes between the sample of men and women (see Figure 1). Findings from the qualitative analysis are discussed below.

### 3.1. Built Environment Barriers 

This sub-theme refers to the influence of the physical features of environments on participation. These include the physical access to spaces as well as features that can support or alternatively dissuade engagement and participation. There were noticeable difference in the frequency of physical environment barriers reported between men and women. Whilst none of the men reported barriers arising from the physical environment, five women participants identified lack of accessibility of public spaces as an environmental barrier that hindered their ability to participate in community-based occupations. Sidewalks and streets in disrepair, lack of accessible parking spaces, and lack of accessibility of public bathrooms were some of the barriers reported by these women. For example, a woman with mild mobility limitations talked about the restrictions arising from perceived lack of safe pedestrian transit areas:

*The sidewalks from this building to the Fatima (community church) are a disaster. They have big holes…, and when it rains it becomes a lake. You know? Very bad, very bad. I used to go, until recently, with a lady.*

*The above expression exemplifies lack of fit between the presence of physical conditions and the environment resulting in restrictions to participate in meaningful occupations. These women also identified lack of space and resources that often characterize senior housing in Puerto Rico which restricted their participation mostly in valuable leisure occupations, as evidenced by the following voice: “I do it less (arts and crafts) because I don’t have space… I can’t continue accumulating things… although you can have whatever you want in your room, forget it. I will have to give up some basic things for another”. In contrast, the physical properties of the environment, such as space limitations or accessibility issues were not perceived restrictors to occupational participation among the men this study.*

### 3.2. Natural Environment Barriers

Natural environment refers to geographical features reported by the participants as a barrier to participation in daily life occupations. Similarities across gender were observed related to the restricting impact of the natural environment to engage in daily occupations. Two men and two women identified the effect of the sun and bright daylight hot weather and rainy conditions of Puerto Rico as challenges that restricted their participation in leisure and social occupations. For example, rainy weather restricted one of the male participant’s ability to drive safely, which in turn constituted a barrier to participation in out-of-home occupations: “When it rains I don't drive. I know myself. Well imagine, at my age killing myself is nothing, but that I kill a human being who has no fault, who’s calm and peaceful and I’m the guilty one...” As seen in this voice, the interaction between weather conditions and social responsibility contributed to participation restrictions.

### 3.3. Social and Economic Systems Restrictions

This sub-theme explains participation barriers at the societal level regarding economic conditions and the availability of governmental services, as well as private or public community resources that support or restrict older adults’ participation in daily occupations. Domains within this sub-theme include *limited community resources, economic restrictions, public transportation challenges,* and *reduced social interaction.*

#### 3.3.1. Limited Community Resources

There were no noticeable differences in reports of limited community resources between men and women. Four men and six women described restricted availability of community resources to support their participation in daily occupations. These participants experienced limited access to community resources needed to engage in community outings, dance clubs, arts and crafts courses, theaters, and community exercise groups. Community resources to participation were not only scarce, but also limitations in public transportation services played a role. As one participant explicitly stated: “The health insurance that I have covers some things (community resources such as physical activity groups and community health activities), but the treatment facilities are all far away and the buses don’t go there.” 

#### 3.3.2. Economic Restrictions

Both men and women of this study similarly reported financial restrictions as a barrier to engage in meaningful occupations, which was not surprising given the low socioeconomic status of the participants of this study. Five women and four men talk about how poor economic condition created limitations to engagement in meaningful daily life tasks, activities, and occupations. For example, economic restraints forced one of the men with mobility limitations to engage in an occupation with lack of meaningfulness: “At the laundries you take the clothes and it cost you $25 or $30. Therefore, you have to try and do it yourself, even if you don’t want to.” Similarly, a woman explained that she could no longer afford to pay for house cleaning services since her husband died: “Before, I used to have someone to help me, but nowadays one cannot pay for cleaning. You know that they are very pricey”. Therefore, economic restrictions lead these individuals to engage in undesired obligatory activities, limiting their repertoire of meaningful and health promoting occupational routines.

#### 3.3.3. Public Transportation Challenges

There were noticeable differences between the men and women from this study in the reported barriers to occupational participation related to limited transportation systems. These barriers were only mentioned by non-driving participants and were predominantly experienced by women (seven) as compared to men (one). Women identified challenges such as lack of physical accessibility, limited geographical areas served by the transportation system, excessive waiting time at bus stops, and lack of bus schedule resulting in inefficient transportation services for older people. For example, a woman with walking difficulties described the lack of fit between the actual design of public buses and the needs of the elderly population: “Transportation here is not for old people. Buses are not equipped to lower the first step down to sidewalk level... this is an enormous problem... they have to think about old age”. This voice demonstrates how restrictions arising from the environment limit the repertoire of occupations that older adults can potentially engage, which had an evident higher impact in the non-driving women of this study.

#### 3.3.4. Reduced Social Interaction

Reduced social interaction was the most frequently cited barrier to continued participation similarly reported by both men and women. Eleven men and 12 women mentioned that reduced social interaction limited their participation in a wide range of significant social occupations, such as attending family meetings, talking on the phone, or having a relationship. Similarly, reduced social interactions hampered these participants’ opportunities to engage in leisure occupations, such as going to restaurants, attending outings or holidays, dancing, and going to the theater or museums. 

The busy lifestyle of participants’ children during their working years, and the experience of bereavement due to losing a partner, also contributed to perceptions of limited social interactions. Participants mentioned reduced opportunities for companionship and also having to take sole responsibility for non-discretionary occupations that are fundamental to support daily life, as evidenced by the voice of the following woman:

*You talk with your companion… you communicate, you share. A mate is needed for many reasons, right? At this time, that problem with fixing the window would have been taken care of by my companion. If my car is not working, if I need something in an emergency, well, my companion gets right on it, do you understand?*

*The loss of friends during old age was another reason given for limited social interaction that then hindered opportunities to participate in meaningful leisure occupations. For example, one man mentioned that he no longer dances because he doesn’t find people available to go dancing with. Even though one of the participants continued to engage in activities that he used to do with others when he was younger, he now experiences reduced pleasure because of the absence of the social component of the occupation: “before I use to go with my daughters (to the movies), now I go alone and it’s more monotonous”.*

Some participants talked about experiencing loneliness due to reduced social interaction. For these participants, living alone was a negative experience that could be a risk factor for depression evidenced by the following voice:

*God said it’s not good for a man to be alone. Older men need someone to come around and check on him. That’s all! To bring him some kind of tea, because they are abandoned and left alone. Just look at him and give him a little conversation. Conversation for an old man means a lot. Loneliness is a punishment. The elderly are alone.*

Overall, the perception of reduced social interaction had a higher impact in restricting participation in social and leisure occupations that are crucial for the experience of wellbeing and life satisfaction.

In summary, women of this study were more likely than men to experience restrictions to participate in meaningful daily activities and occupations due to extrinsic factors related to the built environment and public transportation systems.

## 4. Discussion

The purpose of this study was to explore qualitatively the gender differences regarding environment factors that result in challenges to participation in daily occupations from the perspectives of older people who live alone in Puerto Rico. Understanding environment related challenges that prevent older adults from engaging in meaningful occupations within the complexity of daily lives is critical understanding the forces that shape occupation and how this relates to health and quality of life. Our research adds to the literature by addressing two understudied areas: (1) presenting views of occupational challenges experienced by different gender groups; and (2) representing a group of older adults that have received little attention in Puerto Rico. 

Most of the environment constraining barriers to participation were similarly experienced across gender, including limited community resources, economic restrictions, and reduced social interactions as seen in previous studies with older people who live alone [15,16,17]. However, a new finding of our study reveals some notable differences in perceived occupational challenges across the gender divide. Women were more likely than men to describe challenges to participation in daily life occupations as the result of environmental restrictors. For example, only women participants experienced participation restrictions originated from inaccessible public spaces, a contextual attribute related to infrastructure. This gender difference could be attributed to the societal and cultural gender expectations of roles and occupations. This is, Puerto Rican women have higher levels of participation in out-of-home occupations as compared to men due to having more opportunities participate in occupations traditionally linked to women’s roles and rituals (e.g., shopping and church-based social groups). 

In addition, barriers to access public transportation were also a limitation on participation and were raised by more women as compared to men, as seen in a previous study [28]. Inaccessible public transportation had a higher impact on the women of this study because they were far less likely to own and drive a car. Restricted transportation systems limit the repertoire of occupations that older women can potentially engage. Therefore, women of this study experienced more environmental constraints to access adequate resources to participate in meaningful occupations, putting this population at more risk to experience decreased health and wellbeing. 

In addition to gender differences in the perceived environmental barriers to participation, this study highlighted the emotional impact of experiencing these restrictions. For example, one of the participants used the following expression to describe his experience of living alone: “loneliness is a punishment.” Social isolation not only has a negative impact in emotional wellbeing, but also reduces older people opportunities to receive the amount of social support needed to enable participation in old age. Scientists have long acknowledged the central role of social interactions and involvement in activities to promote better health outcomes [29]. Therefore, those who live alone with reduced social interaction opportunities may be at higher risks to experience a negative effect on health and longevity in older age. 

The findings of this study are of relevance to those working with older men and women experiencing minor disability and living independently at home in the community. This population is an important group to study because of their potential transition to frailer states due restriction imposed by environmental factors. Interventions fostering supportive environments at this stage can play a critical role in delaying, if not transforming, this transition. In addition, our findings highlights the potential 

Our findings suggest several implications for practice, policy, and research. Because of the close relationship between participation, health and wellbeing, healthcare practitioners, social services professionals, and policy makers can use the findings of this study to foster supportive environments to reduce disability. Our results highlight the importance of going beyond the personal level to attend the characteristics of the environment that restricts participation in everyday activities. Specific approaches to foster supportive environments for occupation participation in older Hispanic living in Puerto Rico include skills development in practical strategies to: (1) access free or low-cost local community resources; (2) overcoming obstacles imposed by public transportation systems; and (3) obtaining social support to enable participation in necessary and meaningful occupations.

This study adds to the existing literature regarding the importance of considering the impact of the environment across gender and occupations when delivering occupation-based interventions to older people. Therefore, health care practitioners, social services professionals and policy makers are urged to address the differential impact of the environment in daily participation for women as well as for men to support healthy aging and ensure occupational justice. By facilitating a supportive environment across gender, health and social services professionals may be more effective in enabling older people to engage in health promoting occupations. 

Moreover, findings from this study may serve as empirical evidence when communicating with policy makers. Policies makers responsible for the broader community can develop supportive environments for healthy aging by assuring continuous paths of travel within the community, accessible public transport networks, welcoming accessible senior housings with sufficient space for mobility devices, and supporting community resources and activities for full participation of older people in social, healthy, and productive activities and occupations. In addition, findings of this study suggest that financially supporting elderly who are facing everyday economic difficulties is likely to be beneficial to support participation in daily occupations. These environmental strategies for full participation beyond the home may have a positive impact in older Hispanic state of health because research has shown that supportive social, physical, and economical systems environments can influence older adults wellbeing [5,6,7,8,9,10,11,12,13,30]. 

Health-related public policies in Puerto Rico can be developed and informed by the barriers found in this study to foster supportive environments for participation in occupations that promote individuals’ wellbeing and justice across gender and age.

Limitations of this study include the use of a small, homogeneous, purposive sample that limits its generalizability to Hispanic older adults with similar socio-demographic characteristics. The recruitment of the sample through the use of flyers could also have introduced sampling bias with possible under-representation of less active older adults with higher levels of occupational participation restrictions.

## 5. Conclusions 

This research’s findings provide new understanding of the function of occupation, suggesting that the environment has a differential impact regarding the experience of restrictions in the ability to participate in daily occupations across gender. Environmental barriers to participation represent older peoples’ perceptions and struggles with daily occupations. Additionally, the role of the environment in how it restricts daily occupations, is important to understanding how healthy engagement can be better supported in Hispanic older people. The barriers experienced by the participants of this study are not unique to older Hispanic living in Puerto Rico, but more generally could be applicable to older adults with similar socio-demographic characteristics from a variety of cultures. 

Future studies may use mixed method research to explore how often people participate in meaningful occupations to help inform further the perceived environmental barriers across gender. In addition, results of this study may guide the development and testing of the effectiveness of culturally sensitive occupational-centered programs in the community targeting modifiable environmental restrictors that are sensitive to gender differences. Future studies might also consider using longitudinal experimental design with a representative sample of older adults to examine gender differences in environmental challenges to participate in daily life occupations across samples of older adults with varied living and socio-economic status and ethnicities. 

## Supplementary Files

Supplementary File 1
