# Supplementary material for: Environmental Restrictors to Occupational Participation in Old Age: Exploring Differences across Gender in Puerto Rico
Source: Int J Environ Res Public Health. 2015 Sep 10;12(9):11288–303. doi: 10.3390/ijerph120911288 (PMC4586676; doi:10.3390/ijerph120911288)
Supplement: Supplementary File 1 [file ijerph-12-11288-s001.pdf]

Supplementary Information

Environmental Restrictors to Occupational Participation in Old Age: Exploring Differences across Gender in Puerto Rico

---

Interview Guide

Code number:_____________ **Interviewer:** ________________________

Date: _______________ **Duration of the interview:**_____________

Interview context description:____

____

____

____

Participant's characteristics:

____

____

Preamble: I want to ask you about your experience related to participating in the activities of daily living as an older adult who lives alone. I am particularly interested in what things do you think contribute to experiencing difficulties to engage in the activities that you want to do every day.

Questions	Field Notes
**Meaning of occupation:**<br><br>(1). What are your most important activities of daily living?	
**Experience of occupational challenges**<br><br>(2). What kind of difficulties have you experienced in participating in daily activities?<br><br>(3). What do you think are the barriers or obstacles to do what you want to do every day?<br><br>(4). What is the hardest thing for you as an older adult who lives alone?	

**Closing question:**<br><br>(5) Is there anything else that I should know about the obstacles that you face to participate in your daily life activities?	

© 2015 by the authors; licensee MDPI, Basel, Switzerland. This article is an open access article distributed under the terms and conditions of the Creative Commons Attribution license (http://creativecommons.org/licenses/by/4.0/).
